# 1-(4-Bromo­phen­yl)-3-chloro­propan-1-one

**DOI:** 10.1107/S2414314625006200

**Published:** 2025-07-23

**Authors:** Marcel Sonneck, Anke Spannenberg, Sebastian Wohlrab, Tim Peppel

**Affiliations:** a Leibniz-Institut für Katalyse e V Albert-Einstein-Str 29a 18059 Rostock Germany; Goethe-Universität Frankfurt, Germany

**Keywords:** crystal structure, β-chloro ketone, stacking, halogen bond

## Abstract

The title compound, C_9_H_8_BrClO, crystallizes in the monoclinic space group *P*2_1_/*n* with four mol­ecules in the unit cell. The mol­ecular structure consists of almost planar mol­ecules with the chlorine atom protruding from this plane.

## Abstract 2

The title compound consists of almost planar mol­ecules with the chlorine atom protruding from this plane.

## Graphical abstract

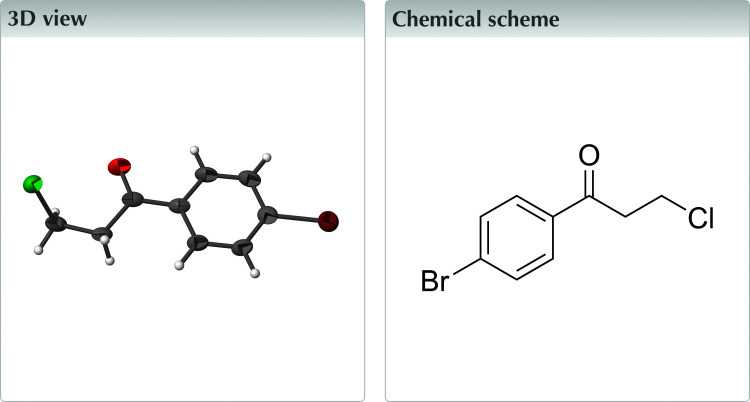

## Structure description

β-Chloro ketones are useful building blocks for many chemical transformation reactions. They are accessible *via* different reactions schemes such as Friedel–Crafts acyl­ation (Sartori & Maggi, 2006 ▸), Wacker-type oxidation (Liu *et al.*, 2017 ▸), or light-mediated ring opening of aryl cyclo­propanes (Petzold *et al.*, 2019 ▸). The title compound was obtained in moderate yield in single-crystal purity. It can be designated as a suitable building block in the ongoing efforts to synthesize feasible new ligands for Cu-based complexes (Sonneck *et al.*, 2015 ▸, 2016 ▸).

**Figure 1:**
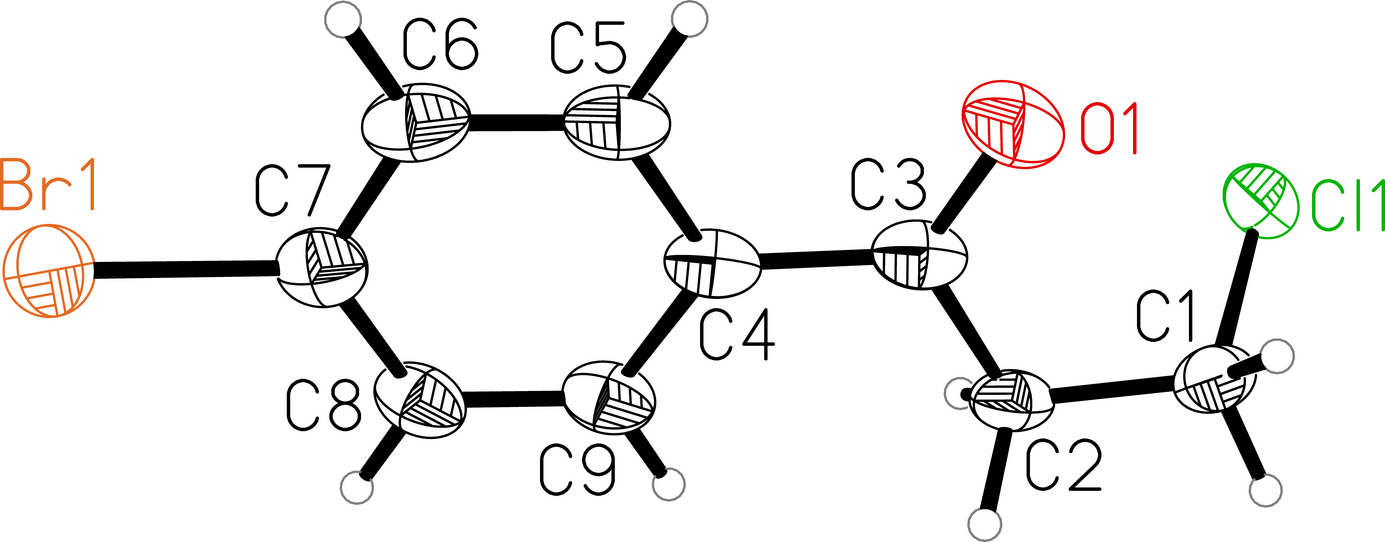
The mol­ecular structure of the title compound with atom labeling and displacement ellipsoids drawn at 50% probability level.

**Figure 2:**
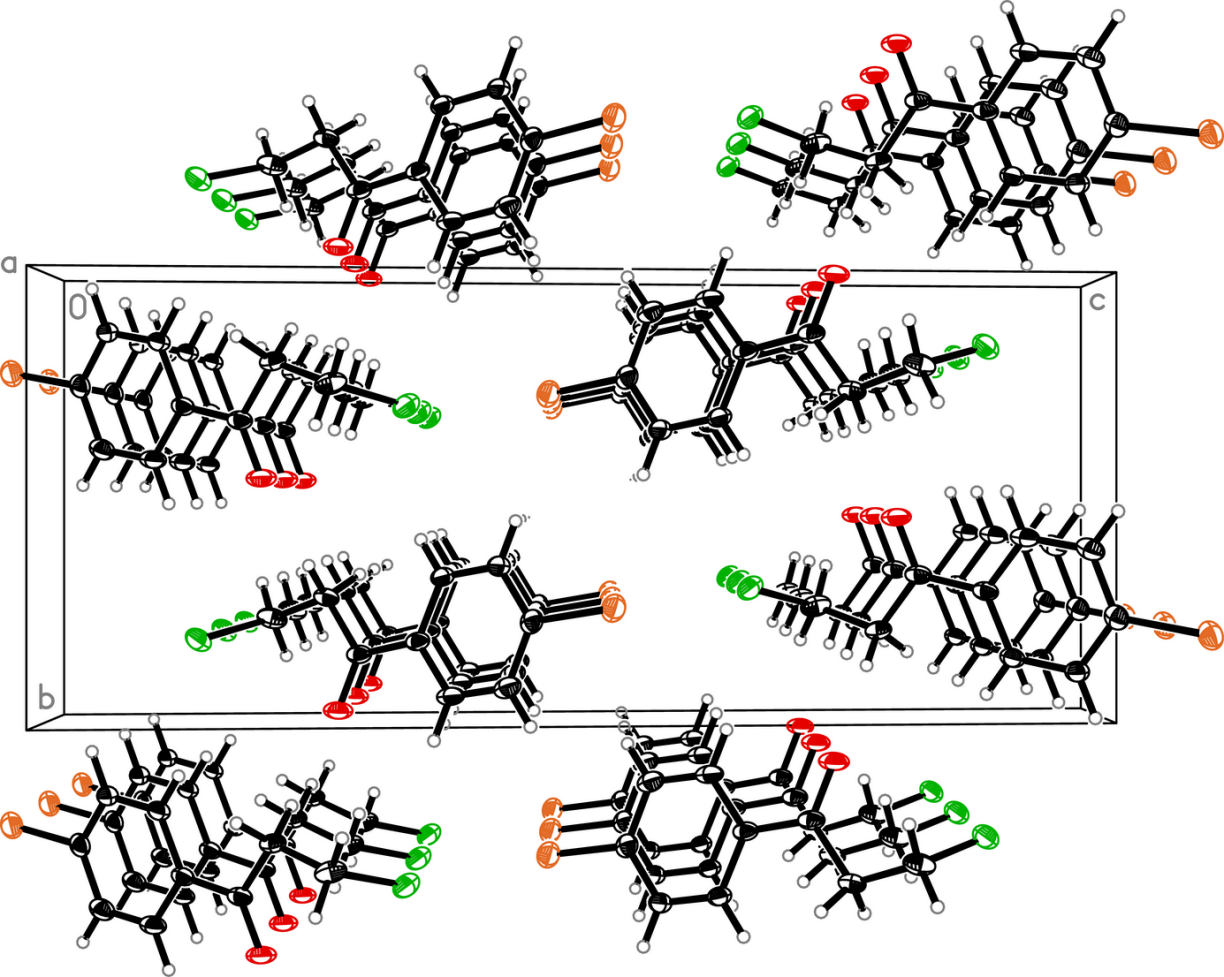
Packing diagram for the title compound along the *a* axis with displacement ellipsoids drawn at 30% probability level.

The mol­ecular structure of the title compound consists of a *para*-substituted bromo­phenyl core and a β-chloro-substituted carbonyl side chain (Fig. 1 ▸). All carbon atoms, Br1 and O1 form a plane with a mean deviation from the best plane defined by these atoms of 0.029 Å. Cl1 is out of that plane by 1.547 (2) Å. All bond lengths and angles are in expected ranges and the C=O bond length is 1.218 (2) Å. The title compound crystallizes in a layered fashion along the crystallographic *a* axis (Fig. 2 ▸). In the crystal, weak C—H⋯O hydrogen bonds link the mol­ecules (Table 1 ▸). The crystal structure is further characterized by type II halogen bonds: Br⋯Cl^i^ = 3.4401 (7) Å; θ_1_: C—Br⋯Cl^i^ = 174.18 (6)°, θ_2_: Br⋯Cl^i^—C^i^ = 103.98 (7)° [symmetry code: (i) *x* + 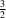, −*y* + 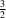, *z* + 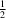] (Metrangolo & Resnati, 2014 ▸).

## Synthesis and crystallization

The title compound was obtained as colorless crystals in moderate yield from the Friedel–Crafts acyl­ation of bromo­benzene and 3-chloro­propionyl chloride in di­chloro­methane. AlCl_3_ (25.5 g, 191.0 mmol, 1.25 eq.) was suspended in 35 ml of di­chloro­methane. A solution of 3-chloro­propionyl chloride (19.4 g, 152.8 mmol, 1.0 eq.) in 10 ml of di­chloro­methane was added dropwise under ambient conditions to the AlCl_3_ suspension and further stirred for 15 min. Afterwards, a solution of bromo­benzene (24.0 g, 152.8 mmol, 1.0 eq.) in 10 ml of di­chloro­methane was added dropwise at room conditions to the suspension and further stirred for 16 h. The final solution was poured onto ice and concentrated hydro­chloric acid (45 g: 15 g). After separation of the organic phase, the aqueous phase was extracted twice with 100 ml portions of di­ethyl­ether. The combined organic phases were extracted once with 150 ml of saturated aqueous Na_2_CO_3_ solution, followed by two extractions with 150 ml portions of water, respectively. The organic phase was finally dried over MgSO_4_ and the solvent was removed completely under diminished pressure. The solid residue was recrystallized from a solvent mixture of hexa­nes to yield a slightly yellowish final product (27.8 g, 74%). Colorless single crystals of C_9_H_8_BrClO were obtained from an acetonic solution by slow evaporation of the solvent at room temperature over the period of one week.

Analytic data for C_9_H_8_BrClO: m.p. 69°C, elemental analysis % (calc.): C 43.85 (43.67), H 3.18 (3.26); Br 32.37 (32.28); Cl 14.19 (14.32). ^1^H NMR (300 MHz, CDCl_3_): δ (p.p.m.) = 7.84–7.79 (*m*, 2H, ArH); 7.65–7.59 (*m*, 2H, ArH); 3.91 (*t*, ^3^*J* = 6.7 Hz, 2H); 3.42 (*t*, ^3^*J* = 6.7 Hz, 2H); ^13^C NMR (75 MHz, CDCl_3_): δ (p.p.m.) = 195.83 (CO); 135.20 (C); 132.22, 132.22, 129.68, 129.68 (CH); 128.96 (C); 41.33, 38.59 (CH_2_).

## Refinement

Crystal data, data collection and structure refinement details are summarized in Table 2 ▸.

## Supplementary Material

Crystal structure: contains datablock(s) I. DOI: 10.1107/S2414314625006200/bt4177sup1.cif

Structure factors: contains datablock(s) I. DOI: 10.1107/S2414314625006200/bt4177Isup2.hkl

Supporting information file. DOI: 10.1107/S2414314625006200/bt4177Isup3.cml

CCDC reference: 1495202

Additional supporting information:  crystallographic information; 3D view; checkCIF report

## Figures and Tables

**Table 1:** Hydrogen-bond geometry (Å, °)

*D*—H⋯*A*	*D*—H	H⋯*A*	*D*⋯*A*	*D*—H⋯*A*
C1—H1*A*⋯O1^i^	0.99	2.31	3.176 (2)	146
C2—H2*B*⋯O1^ii^	0.99	2.54	3.535 (2)	178

Symmetry codes: (i) 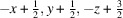; (ii) 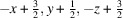.

**Table 2:** Experimental details

Crystal data	
Chemical formula	C_9_H_8_BrClO
*M*_r_	247.51
Crystal system, space group	Monoclinic, *P*2_1_/*n*
Temperature (K)	150
*a*, *b*, *c* (Å)	4.4730 (7), 9.3707 (15), 22.366 (4)
β (°)	94.989 (3)
*V* (Å^3^)	933.9 (3)
*Z*	4
Radiation type	Mo *K*α
μ (mm^−1^)	4.63
Crystal size (mm)	0.42 × 0.30 × 0.12

Data collection	
Diffractometer	Bruker APEXII CCD
Absorption correction	Multi-scan (*SADABS*; Krause *et al.*, 2015 ▸)
*T*_min_, *T*_max_	0.41, 0.60
No. of measured, independent and observed [*I* > 2σ(*I*)] reflections	9215, 2037, 1780
*R*_int_	0.026
(sin θ/λ)_max_ (Å^−1^)	0.639

Refinement	
*R*[*F*^2^ > 2σ(*F*^2^)], *wR*(*F*^2^), *S*	0.023, 0.058, 1.03
No. of reflections	2037
No. of parameters	109
H-atom treatment	H-atom parameters constrained
Δρ_max_, Δρ_min_ (e Å^−3^)	0.37, −0.37

Computer programs: *APEX2* (Bruker, 2014 ▸), *SAINT* (Bruker, 2013 ▸), *SHELXS97* (Sheldrick, 2008 ▸), *SHELXL* (Sheldrick, 2015 ▸), *XP* in *SHELXTL* (Sheldrick, 2008 ▸) and *publCIF* (Westrip, 2010 ▸).

## full crystallographic data

### 1-(4-Bromophenyl)-3-chloropropan-1-one Crystal data

**Table:**

C_9_H_8_BrClO	*F*(000) = 488
*M**_r_* = 247.51	*D*_x_ = 1.760 Mg m^−^^3^
Monoclinic, *P*2_1_/*n*	Mo *K*α radiation, λ = 0.71073 Å
*a* = 4.4730 (7) Å	Cell parameters from 4023 reflections
*b* = 9.3707 (15) Å	θ = 2.2–28.0°
*c* = 22.366 (4) Å	µ = 4.63 mm^−^^1^
β = 94.989 (3)°	*T* = 150 K
*V* = 933.9 (3) Å^3^	Plate, colourless
*Z* = 4	0.42 × 0.30 × 0.12 mm

### 1-(4-Bromophenyl)-3-chloropropan-1-one Data collection

**Table:**

Bruker APEXII CCD diffractometer	2037 independent reflections
Radiation source: fine-focus sealed tube	1780 reflections with *I* > 2σ(*I*)
Detector resolution: 8.3333 pixels mm^-1^	*R*_int_ = 0.026
φ and ω scans	θ_max_ = 27.0°, θ_min_ = 1.8°
Absorption correction: multi-scan (SADABS; Krause *et al.*, 2015)	*h* = −5→5
*T*_min_ = 0.41, *T*_max_ = 0.60	*k* = −11→11
9215 measured reflections	*l* = −28→27

### 1-(4-Bromophenyl)-3-chloropropan-1-one Refinement

**Table:**

Refinement on *F*^2^	Primary atom site location: structure-invariant direct methods
Least-squares matrix: full	Hydrogen site location: inferred from neighbouring sites
*R*[*F*^2^ > 2σ(*F*^2^)] = 0.023	H-atom parameters constrained
*wR*(*F*^2^) = 0.058	*w* = 1/[σ^2^(*F*_o_^2^) + (0.028*P*)^2^ + 0.3453*P*] where *P* = (*F*_o_^2^ + 2*F*_c_^2^)/3
*S* = 1.03	(Δ/σ)_max_ = 0.004
2037 reflections	Δρ_max_ = 0.37 e Å^−^^3^
109 parameters	Δρ_min_ = −0.37 e Å^−^^3^
0 restraints	

### 1-(4-Bromophenyl)-3-chloropropan-1-one Special details

**Table:**

Geometry. All esds (except the esd in the dihedral angle between two l.s. planes) are estimated using the full covariance matrix. The cell esds are taken into account individually in the estimation of esds in distances, angles and torsion angles; correlations between esds in cell parameters are only used when they are defined by crystal symmetry. An approximate (isotropic) treatment of cell esds is used for estimating esds involving l.s. planes.
Refinement. Hydrogen atoms were located in a difference map and refined as riding on their parent atoms with *U*(H)=1.2*U*_eq_(C) and with C_aromatic_—H=0.95 Å or with C_methylene_—H=0.99 Å.

### 1-(4-Bromophenyl)-3-chloropropan-1-one Fractional atomic coordinates and isotropic or equivalent isotropic displacement parameters (Å^2^)

**Table:**

	*x*	*y*	*z*	*U*_iso_*/*U*_eq_	
Br1	1.42604 (5)	0.77477 (2)	1.02881 (2)	0.04497 (9)	
C1	0.2245 (5)	0.7340 (2)	0.71439 (11)	0.0405 (5)	
H1A	0.077806	0.810098	0.702401	0.049*	
H1B	0.111428	0.648575	0.725442	0.049*	
C2	0.4251 (4)	0.78300 (19)	0.76779 (9)	0.0323 (4)	
H2A	0.301478	0.831498	0.796299	0.039*	
H2B	0.569695	0.853619	0.754374	0.039*	
C3	0.5973 (4)	0.66257 (19)	0.80011 (9)	0.0326 (4)	
C4	0.7958 (4)	0.69450 (19)	0.85496 (9)	0.0317 (4)	
C5	0.9448 (4)	0.5819 (2)	0.88552 (10)	0.0391 (5)	
H5	0.916508	0.487417	0.870675	0.047*	
C6	1.1317 (5)	0.6049 (2)	0.93664 (10)	0.0409 (5)	
H6	1.231700	0.527279	0.957094	0.049*	
C7	1.1722 (5)	0.7430 (2)	0.95792 (9)	0.0354 (4)	
C8	1.0295 (5)	0.8566 (2)	0.92856 (9)	0.0369 (4)	
H8	1.060492	0.950893	0.943426	0.044*	
C9	0.8415 (4)	0.8323 (2)	0.87750 (9)	0.0356 (4)	
H9	0.741597	0.910338	0.857366	0.043*	
Cl1	0.43714 (12)	0.69179 (5)	0.65212 (2)	0.04357 (14)	
O1	0.5732 (3)	0.54110 (14)	0.78086 (7)	0.0461 (4)	

### 1-(4-Bromophenyl)-3-chloropropan-1-one Atomic displacement parameters (Å^2^)

**Table:**

	*U*^11^	*U*^22^	*U*^33^	*U*^12^	*U*^13^	*U*^23^
Br1	0.05104 (15)	0.04894 (15)	0.03549 (13)	0.00654 (9)	0.00697 (9)	0.00511 (9)
C1	0.0265 (9)	0.0303 (10)	0.0646 (14)	0.0023 (8)	0.0027 (9)	−0.0085 (9)
C2	0.0295 (9)	0.0223 (9)	0.0462 (11)	0.0010 (7)	0.0102 (8)	−0.0044 (8)
C3	0.0336 (9)	0.0220 (9)	0.0444 (11)	−0.0017 (7)	0.0162 (8)	0.0018 (8)
C4	0.0362 (10)	0.0219 (9)	0.0394 (10)	0.0001 (7)	0.0168 (8)	0.0037 (7)
C5	0.0461 (11)	0.0209 (9)	0.0517 (13)	0.0034 (8)	0.0122 (10)	0.0036 (8)
C6	0.0462 (11)	0.0304 (10)	0.0478 (12)	0.0090 (8)	0.0129 (10)	0.0093 (9)
C7	0.0377 (10)	0.0380 (11)	0.0323 (10)	0.0027 (8)	0.0138 (8)	0.0046 (8)
C8	0.0519 (12)	0.0258 (9)	0.0344 (10)	0.0018 (8)	0.0116 (9)	0.0000 (8)
C9	0.0479 (11)	0.0230 (9)	0.0371 (11)	0.0034 (8)	0.0112 (9)	0.0043 (8)
Cl1	0.0478 (3)	0.0371 (3)	0.0442 (3)	0.0059 (2)	−0.0056 (2)	−0.0114 (2)
O1	0.0597 (9)	0.0189 (7)	0.0595 (10)	−0.0032 (6)	0.0031 (7)	−0.0020 (6)

### 1-(4-Bromophenyl)-3-chloropropan-1-one Geometric parameters (Å, º)

**Table:**

Br1—C7	1.892 (2)	C4—C9	1.394 (3)
C1—C2	1.502 (3)	C4—C5	1.395 (3)
C1—Cl1	1.798 (2)	C5—C6	1.374 (3)
C1—H1A	0.9900	C5—H5	0.9500
C1—H1B	0.9900	C6—C7	1.385 (3)
C2—C3	1.514 (3)	C6—H6	0.9500
C2—H2A	0.9900	C7—C8	1.378 (3)
C2—H2B	0.9900	C8—C9	1.377 (3)
C3—O1	1.218 (2)	C8—H8	0.9500
C3—C4	1.481 (3)	C9—H9	0.9500
			
C2—C1—Cl1	111.33 (14)	C5—C4—C3	118.65 (17)
C2—C1—H1A	109.4	C6—C5—C4	121.30 (18)
Cl1—C1—H1A	109.4	C6—C5—H5	119.4
C2—C1—H1B	109.4	C4—C5—H5	119.4
Cl1—C1—H1B	109.4	C5—C6—C7	118.98 (18)
H1A—C1—H1B	108.0	C5—C6—H6	120.5
C1—C2—C3	113.32 (16)	C7—C6—H6	120.5
C1—C2—H2A	108.9	C8—C7—C6	121.1 (2)
C3—C2—H2A	108.9	C8—C7—Br1	119.80 (15)
C1—C2—H2B	108.9	C6—C7—Br1	119.09 (15)
C3—C2—H2B	108.9	C9—C8—C7	119.43 (18)
H2A—C2—H2B	107.7	C9—C8—H8	120.3
O1—C3—C4	120.65 (17)	C7—C8—H8	120.3
O1—C3—C2	120.12 (18)	C8—C9—C4	120.85 (18)
C4—C3—C2	119.23 (16)	C8—C9—H9	119.6
C9—C4—C5	118.32 (19)	C4—C9—H9	119.6
C9—C4—C3	123.02 (17)		
			
Cl1—C1—C2—C3	−75.11 (19)	C4—C5—C6—C7	−0.1 (3)
C1—C2—C3—O1	2.5 (3)	C5—C6—C7—C8	−0.2 (3)
C1—C2—C3—C4	−177.81 (16)	C5—C6—C7—Br1	179.31 (15)
O1—C3—C4—C9	177.21 (18)	C6—C7—C8—C9	0.6 (3)
C2—C3—C4—C9	−2.4 (3)	Br1—C7—C8—C9	−179.01 (15)
O1—C3—C4—C5	−2.6 (3)	C7—C8—C9—C4	−0.5 (3)
C2—C3—C4—C5	177.73 (17)	C5—C4—C9—C8	0.1 (3)
C9—C4—C5—C6	0.2 (3)	C3—C4—C9—C8	−179.70 (17)
C3—C4—C5—C6	−179.99 (18)		

### 1-(4-Bromophenyl)-3-chloropropan-1-one Hydrogen-bond geometry (Å, º)

**Table:**

*D*—H···*A*	*D*—H	H···*A*	*D*···*A*	*D*—H···*A*
C1—H1*A*···O1^i^	0.99	2.31	3.176 (2)	146
C2—H2*B*···O1^ii^	0.99	2.54	3.535 (2)	178

Symmetry codes: (i) −*x*+1/2, *y*+1/2, −*z*+3/2; (ii) −*x*+3/2, *y*+1/2, −*z*+3/2.
